# Burnout’s in young doctors: Prevalence, socio-demographic and psychological associated factors

**DOI:** 10.1192/j.eurpsy.2021.1520

**Published:** 2021-08-13

**Authors:** S. Kolsi, S. Hentati, I. Baati, J. Masmoudi

**Affiliations:** Psychiatry A, CHU Hédi Chaker Sfax, Sfax, Tunisia

## Abstract

**Introduction:**

Burn-out is quite common in hospitals especially among young doctors. It results from a mismatch between expectation and professional reality.

**Objectives:**

To determine the prevalence of sever burnout and to identify its associated socio-demographic and psychological factors among young residents.

**Methods:**

Analytical and descriptive cross-sectional study conducted among residents and interns working at the Hedi Chaker and Hbib Bourguiba University hospital in Sfax, Tunisia, during the month of July 2019. The characteristics of the participants were collected using a questionnaire. Burnout was evaluated using the Maslach Burnout Inventory(MBI) differentiating 3 components: emotional exhaustion, depersonalization and luck of personal achievement.

**Results:**

Out of 85 questionnaires disturbed,60 were selected corresponding to a response rate of 72.94%. The sex ratio (M/F) was 0.87. The middle age was 28.22. Forty three percent of the participants were married. More than half consumed tobacco and 45% of them consumed alcohol. The majority of doctors were residents(81.7%). The average working time was 55 hours per week. Burn-out was severe in 30% of our population. Furthermore, doctors who suffered from physical aggression(p=0.001) were more likely to develop severe burn-out. The dissatisfaction with the internship (p=0.01) and the feeling of do not satisfy seniors(p=0.02) were statistically associated with severe burnout. Severe burn-out was associated with anxiety (p=0.0073), conflictual partnership (p=0.0001),conflicts with colleagues(p=0.001) and the paramedical framework (p=0.0001)

**Conclusions:**

The risk of burn-out is quite high among young doctors. Some factors seem to be associated with this phenomenon. This could affect not only the quality of life, but also the quality of care provided.

**Keyword:**

burnout-young doctors-prevalence-associated factors

